# The urban in ecology: a quantitative textual analysis of the scientific literature over a century

**DOI:** 10.1007/s11252-024-01603-4

**Published:** 2024-09-06

**Authors:** Silvia Flaminio, Joëlle Salomon Cavin, Guillaume Guex, Marco Moretti

**Affiliations:** 1 Université de Lausanne, Faculté des Géosciences et de l’Environnement, Institut de Géographie et Durabilité, Géopolis, 1015 Lausanne, Switzerland; 2 Present Address: École Normale Supérieure de Lyon, 15 Parvis René Descartes, BP 7000, 69342 Cedex 07 Lyon, France; 3 Section des Sciences du Langage et de l’information, Université de Lausanne, Faculté des Lettres, Anthropole, 1015 Lausanne, Switzerland; 4 Snow and Landscape Research WSL, Biodiversity and Conservation Biology, Swiss Federal Institute for Forest, Zürcherstrasse 111, 8903 Birmensdorf, Switzerland

**Keywords:** Bibliometrics, Historical trend, Quantitative textual data analysis, Urban ecology

## Abstract

Many authors have described ‘urban ecology’ as a subfield of ecology that emerged in the 1990s and has expanded exponentially. We propose to take a step further and analyse the expansion of the ‘urban’ in ecology with a novel quantitative approach, with the aim to better understand the relationship between ecology and the urban. Previous quantitative assessments of the urban in ecology have focused on short to medium time spans (5 to 40 years) and on research coined as ‘urban ecology’, and have rarely considered the content of publications (e.g., vocabulary and topics) using quantitative methods. In this paper, we conduct a bibliometric assessment and an in-depth quantitative textual data analysis of a corpus of 960 articles published from 1922 to 2018 in 10 leading English-language journals in ecology and conservation biology. We address the following questions: (1) When and how have urban environments been integrated into ecological research during the past century? (2) What urban research topics have been investigated in ecology during the same period? (3) How have these research topics changed through time? Our results show that the urban was never entirely absent from publications in ecology. The quantitative analyses highlight three turning points (1970s, 1990s and 2000s) in the relationship between ecology and the urban. Moreover, they help visualize the shift from particularly scattered publications at the beginning of the period to publications characterized by a more homogeneous vocabulary, reflecting the stabilization of a research field focused on the urban in ecology.

**Supplementary Information:**

The online version contains supplementary material available at 10.1007/s11252-024-01603-4.

## Introduction

Research on the ‘urban’ in ecology is often coined as ‘urban ecology’, a subfield of ecology that can be defined as the study of factors influencing the distribution and abundance of organisms in urban environments and the processes resulting from their interactions with the environment (Pickett et al. 2011). Urban ecology emerged in the 1970s, with the creation, for example, of a dedicated journal (*Urban Ecology*, created in 1975 by urban planner LaNier and merged with *Landscape and Urban Planning* in 1986), and has undergone a boom in recent decades (McDonnell 2015; Barot et al. 2019; Tan 2020). Nonetheless, prior to the emergence of urban ecology, pioneering studies were conducted on cities and urban areas (Kühnelt 1955; Kieran 1959; Sukopp 1973; Duvigneaud 1974; Kunick 1974).

A key marker of the increasing interest of ecologists in the urban environment is the publication trend through time, as underlined in previous reviews in ecology (e.g., Barot et al. 2019). Indeed, until the 1990s, urban ecology was hardly present in international journals (e.g., McDonnell et al. 2011; Wu 2014). By contrast, the end of the 1990s and the 2000s have been characterized by the creation of new academic journals dedicated to urban ecology (e.g., *Urban Ecosystems* in 1997, *Journal of Urban Ecology* and *Frontiers in Urban Ecology* in 2015), the rise of digital forums (e.g., *The Nature of Cities* in 2012) and the publication of review articles and textbooks (e.g., Alberti 2008; Marzluff et al. 2008; Niemelä 2011; Forman 2014; Douglas et al. 2020).

So far, only a few studies have involved long-term quantitative assessments of the evolution of publications dealing with the urban in ecology; to our knowledge, only five papers have had such a focus (Collins et al. 2000; Miller and Hobbs 2002; Young and Wolf 2006; Martin et al. 2012; Barot et al. 2019). The quantitative assessments in these five articles were largely based on the computation of the percentage of papers focusing on cities. For example, Collins et al. (2000) highlighted that from 1995 to 2000 “a mere 0.4 percent – 25 of 6,157 – of the papers published in nine leading ecological journals […] dealt with cities or urban species” (p. 416). Miller and Hobbs (2002) found that less than 6% of the papers published in *Conservation Biology* described studies of human settlements (urban, suburban and exurban). Martin et al. (2012) reviewed 2,573 papers from 10 highly cited ecology journals from 2004 to 2009 and concluded that 3.9% of the study sites were in ‘densely settled’ areas. More recently, Barot et al. (2019) conducted a literature review combining the terms ‘urban’ and ‘ecology’ for the period 1980–2015 in Web of Science and reported that 14,000 articles were published each year on urban ecology, which represents 14% of the total number of articles published in ecology, with an exponential increase of publications in urban ecology. Such assessments are interesting, yet they mostly focused on short to medium time spans (from 5 to 40 years) and provide little information on the content of the publications on urban settings. Young and Wolf (2006) looked into the content of publications in urban ecology and focused on goal attainment and commitments in urban ecology, based on an analysis of all the articles published in two journals (*Urban Ecology* and *Urban Ecosystems*) between 1975 and 2004 (*n* = 261). However, this in-depth quantitative study of urban ecology was only based on papers published in urban ecology journals. Such an approach is relevant for exploring the subfield of urban ecology and its specificities, but it leaves aside publications on the urban by authors who may not anchor their work in the subfield of urban ecology. Finally, all four of these literature reviews mostly adopted a bibliometric approach, and none of them explored in depth and from a quantitative perspective the content, and notably the vocabulary, used in the publications and the topics developed in relation to cities.

The overall aim of this article is to provide a novel contribution to the understanding of the rise of the urban in ecology, and more generally of the relationship between ecology and the urban, thus helping to fill three research gaps: (1) the lack of quantitative analysis over a century; (2) the exclusion of articles which do not label themselves as ‘urban ecology’ yet may still focus on (or include) urban areas; (3) the lack of quantitative assessments regarding the content, the topics and notably the vocabulary used in scientific publications in ecology and on the urban. We ask the following questions:

- When and how have urban environments been integrated into ecological research during the past century?
- What urban research topics have been investigated in ecology during the same period?
- How have these urban research topics changed through time?

While this study examines the case of the urban in ecology and the rise of urban ecology, the implications are far broader. The case study presents an opportunity to engage in the literature on discipline and specialty formation. In the science and technology literature, and especially in the field of ‘new political sociology of science’, these processes are mainly studied through qualitative analysis. For example, studies show how scientists and researchers manage to incorporate certain research problems and issues into broader political and scientific agendas (Frickel 2004) or address researchers’ different views of which knowledge matters most (Granjou and Arpin 2015; Granjou et al. 2023). By conducting a bibliometric analysis and textual data analysis on a substantial corpus of publications in the field, we aim to scrutinize how the vocabulary used in publications in ecology has changed over time. We further aim to show how changes in vocabulary reflect the weak signals of an interest for a new subject (here, the urban) in a discipline and the change in this interest. Finally, we aim to identify key time points in the conceptualization and definition, or even institutionalization, of a new subfield of research.

In the next section we detail our framework and methodology, which combines bibliometrics and textual data analysis. We then present our main results, and more specifically the main trends that we have identified. Finally, we discuss the contribution of our results with regard to previous qualitative reviews on the relationship between ecology and the urban and on the history of urban ecology, and we present the limitations of our quantitative textual data analysis.

## Methods

### General approach

In this paper, we apply bibliometric methods and quantitative textual data analysis to understand how urban environments have been integrated into ecological research and to explore the lexical content of publications on cities in ecology.

Bibliometric studies have been developed in scientometrics “to shed light on the processes of written communication and of the nature and course of development of a discipline (in so far as this is displayed through written communication), by means of counting and analyzing the various facets of written communication” (Pritchard 1969). Bibliometric studies often rely on quantitative citation analysis to investigate the emergence and development of a research field or topic. However, bibliometrics do not focus on the content of the publications, on the words used in the publications, or on the statements made by the authors of the articles. For this reason, in this paper we combine bibliometrics with quantitative textual data analysis.

Textual data analysis, sometimes also called ‘text mining’, can be defined as a set of methods that use statistics to analyse text corpora (Lebart et al. 1998, 2019; Heiden et al. 2010; Beaudouin 2016). Recently, quantitative textual data analysis has been used to explore the textual content of scientific publications and their evolution in time based on the analysis of corpora of abstracts (Dufour et al. 2019; Desvallées et al. 2022) and in some cases of full-length publications (Flaminio et al. 2022a, b). Such methods, which stem from linguistics rather than from scientometrics, are particularly efficient to detect historical trends and to highlight similarities and contrasts in vocabulary and topics; they can be used to characterize phases in the development of research fields (Dufour et al. 2019). Gobster (2014) uses text mining methods, for example, to explore the themes and trends of the papers published in *Landscape and Urban Planning*.

### Building a corpus of scientific papers on the urban in ecology

Text corpora can be defined as collections of texts (or possibly of other media such as pictures of videos) which are put together with specific hypothesis in mind (Mayaffre 2002). We built a coherent and homogeneous corpus (Pincemin 1999) by focusing our study on scientific articles, since they present the advantage of having similar lengths and structures. Since our scope was the evolution and trajectory of publications on the urban in ecology, we focused on journals that are among the oldest available online and that were all founded before 1990. This led to the selection of ten ecology journals, eight with a broad perspective and two focused on conservation biology (Table 1). We also chose the latter because they published important papers related to the cities and conservation (notably Miller and Hobbs 2002; Sanderson and Huron 2011). Data collection was performed using the Web of Science, JSTOR and ScienceDirect databases, as the last two databases are more complete for the period 1900–1990.

**Table 1:** Journals selected for this study. The scope of each journal is reported. The total number of articles from the journal’s foundation to December 2018 is based on data from Web of Science

Journal name	Current scope of the journal extracted from its website	First issue	Number of articles included in the corpus	Number of articles published since the journal’s foundation	Percentage of articles included in the corpus per journal
*Journal of Ecology*	“*Journal of Ecology* [a journal of the British Ecological Society] publishes original research papers on all aspects of the ecology of plants (including algae), in both aquatic and terrestrial ecosystems.”	1913	25	6,548	0.38%
*Ecology*	“*Ecology* [a journal of the Ecological Society of America] publishes research and synthesis papers on all aspects of ecology, with particular emphasis on papers that develop new concepts in ecology, that test ecological theory, or that lead to an increased appreciation for the diversity of ecological phenomena.”	1920	72	16,269	0.44%
*Ecological Monographs*	“Papers published in *Ecological Monographs* [a journal of the Ecological Society of America] provide integrative and complete documentation of major empirical and theoretical advances in the field and establish benchmarks from which future research will build.”	1931	7	1,543	0.45%
*Journal of Animal Ecology*	“*Journal of Animal Ecology* [a journal of the British Ecological Society] publishes the best animal ecology research that develops, tests and advances broad ecological principles.”	1932	55	5,731	0.95%
*Oikos*	“*Oikos* [a journal of the Nordic Society Oikos] publishes original and innovative research on all aspects of ecology, defined as organism-environment interactions at various spatiotemporal scales, so including macroecology and evolutionary ecology”	1949	38	8,715	0.43%
*Journal of Applied Ecology*	“*Journal of Applied Ecology* [a journal of the British Ecological Society] publishes novel, high-impact papers on the interface between ecological science and the management of biological resources.”	1964	111	5,639	2.07%
*Biological Conservation*	“*Biological Conservation* [an affiliate publication of the Society for Conservation Biology] is a leading international journal in the discipline of conservation science. The journal publishes articles spanning a diverse range of fields that contribute to the biological, sociological, ethical and economic dimensions of conservation.”	1968	350	8,291	4.22%
*Oecologia*	“*Oecologia* [published in cooperation with the International Association for Ecology] publishes innovative ecological research of general interest to a broad international audience. We publish several types of manuscripts in many areas of ecology.”	1968	74	12,905	0.57%
*Trends in Ecology and Evolution*	“*Trends in Ecology and Evolution* contains polished, concise and readable reviews, opinions and letters in all areas of ecology and evolutionary science. It serves as an invaluable source of information for researchers, lecturers, teachers, field workers and students.”	1986	24	4,770	0.50%
*Conservation Biology*	“*Conservation Biology* [a journal of the Society for Conservation Biology] is the most influential and frequently cited journal in its field. The journal publishes groundbreaking papers and is instrumental in defining the key issues contributing to the science and practice of conserving Earth’s biological diversity.”	1987	204	5,465	3.73%

We built a broad query, to be as inclusive as possible, using the following combination of words: ‘urban* OR city OR suburb* OR town OR “residential area” OR “human settlement” OR “built environment” OR street’ (in singular and plural forms). We applied this query to titles, keywords and abstracts in the three databases and for 10 selected journals. This resulted in over 4,641 papers before duplicate suppression. By default, we decided to include in the corpus all articles containing ‘urban’ or ‘city’ in their titles or abstracts (n = 676). To exclude off-topic papers, we read the remaining papers; we excluded from the corpus all articles that only marginally mentioned urban areas or urbanization. We ultimately kept a total of 960 papers published between 1922 and December 2018.

We collected the articles in *.pdf format and batch converted them to text files (using the open-source command line utility *pdftotext* and the online tool *pdf2go* when the characters were not recognized in the *.pdf file). Finally, using the open-source programs Regexxer and Notepad +  + , we semi-automatically erased from the articles any information that risked biasing the textual data analysis or interrupting the text structure: authors names, journal names, page numbers, repetitions of the paper title, reference lists, acknowledgements, tables, and figure and table captions.

Once we had finalized the corpus, we built a metadata table containing the following information on each article: the publication date, the authors, and the journal in which the article was published.

### Data analysis

First, from a bibliometric perspective, we determined whether there were publications on the urban in ecology before the 1990s, and we plotted the number of articles published per year in the 10 journals using R (R Core Team 2022). Second, to identify the research themes on which the papers focus, we conducted a textual data analysis on the corpus.

To carry out the textual data analysis we used the open-source software Iramuteq (an R interface for multidimensional analyses of texts and questionnaires; (Ratinaud and Déjean 2009). This software has been used in various publications to analyse survey results (e.g., Torres et al. 2018), and to explore the scientific literature on specific topics (e.g., Hamman 2017), mostly based on corpora of article abstracts (Plumecocq 2014; Allain et al. 2017; Dufour et al. 2019; Curt 2021). In the latter case, the results from Iramuteq are often used to interpret trends in the evolution of scientific fields and topics.

Iramuteq relies on R and the Python programming language (Van Rossum and Drake 1995) to perform its clustering algorithm (Reinert 1983, 1990), which comprises five main phases (Cottet et al. 2015). (1) The clustering algorithm identifies active words (i.e., words potentially carrying a semantic value) and splits the text into segments containing a constant number of active words (which corresponds, in our case, to approximatively 40 words per segment). (2) The algorithm then lemmatizes the words using a grammatical dictionary. In linguistics, a lemma is generally defined as “a set of lexical forms having the same stem and belonging to the same major word class, differing only in inflection and/or spelling” (Francis and Kučera 1982, p. 1). In general, a lemma is the standard dictionary entry of a word. To lemmatize means to tag words of a corpus according to their stem. In our case, the software matched each word of the corpus to a dictionary entry. The algorithm then (3) produces a contingency table between the lemmas and text segments, and (4) performs a top-down hierarchical spectral clustering method on text segments, using the χ^2^ distance computed with the contingency table. The top-down approach means that all text segments start in the same cluster and are then divided recursively according to their first factorial coordinate (obtained with a correspondence analysis). For a complete description of the method see Reinert (1993). (5) The algorithm finally computes the signed χ^2^ association metric (Reinert 1993) between active lemmas and resulting clusters. Reinert defines the signed χ^2^ association metric between two modalities (belonging to two different categorical variables) as the value of the χ^2^ statistic test of the 2 × 2 contingency table with both modalities versus the rest. This statistic is signed based on whether the modalities attract (positive) or repel (negative) each other. It is therefore possible to compute the degree of association between these semantic fields and any corpus metadata, such as the publishing date (again by using the signed χ^2^ association metric). We tested several levels of hierarchical spectral clustering, and the results with 10 clusters (i.e., 10 semantic fields) offered a good balance between lexical diversity, interpretability, and a relatively small number of groups. We characterized each cluster in a similar way to in previous studies (e.g., Desvallées et al. 2022), using Iramuteq to identify text segments and articles which were strongly associated with the clusters.

To understand the evolution in time of these ten semantic fields, we conducted two analyses in parallel. First, using Python, we computed and plotted the signed χ^2^ association metric values (with dotted lines representing the 0.01 significance level for a χ^2^ distribution with 1 degree of freedom, which is equal to 6.63) representing the over- and underrepresentation of the different semantic fields among publication years. Second, we conducted a correspondence analysis based on the contingency table between lemmas appearing at least 1,000 times in the corpus and publication years. On the resulting factorial map, we displayed: (1) the top 10 lemmas for each cluster (regarding their χ^2^ associations), with size of the text corresponding to the frequency in the corpus; (2) triangles: the barycentres, or the centres of mass, of the semantic fields, computed as the weighted (by the positive χ^2^ associations) average of the lemma coordinates; (3) red dots: publication years, with dot size corresponding to the aggregated number of lemmas; and (4) blue line: the ± 2-year moving average around the displayed year, indicating the general trend of the articles over time.

## Results

### Overview of the distribution of the publications

**Fig. 1:**
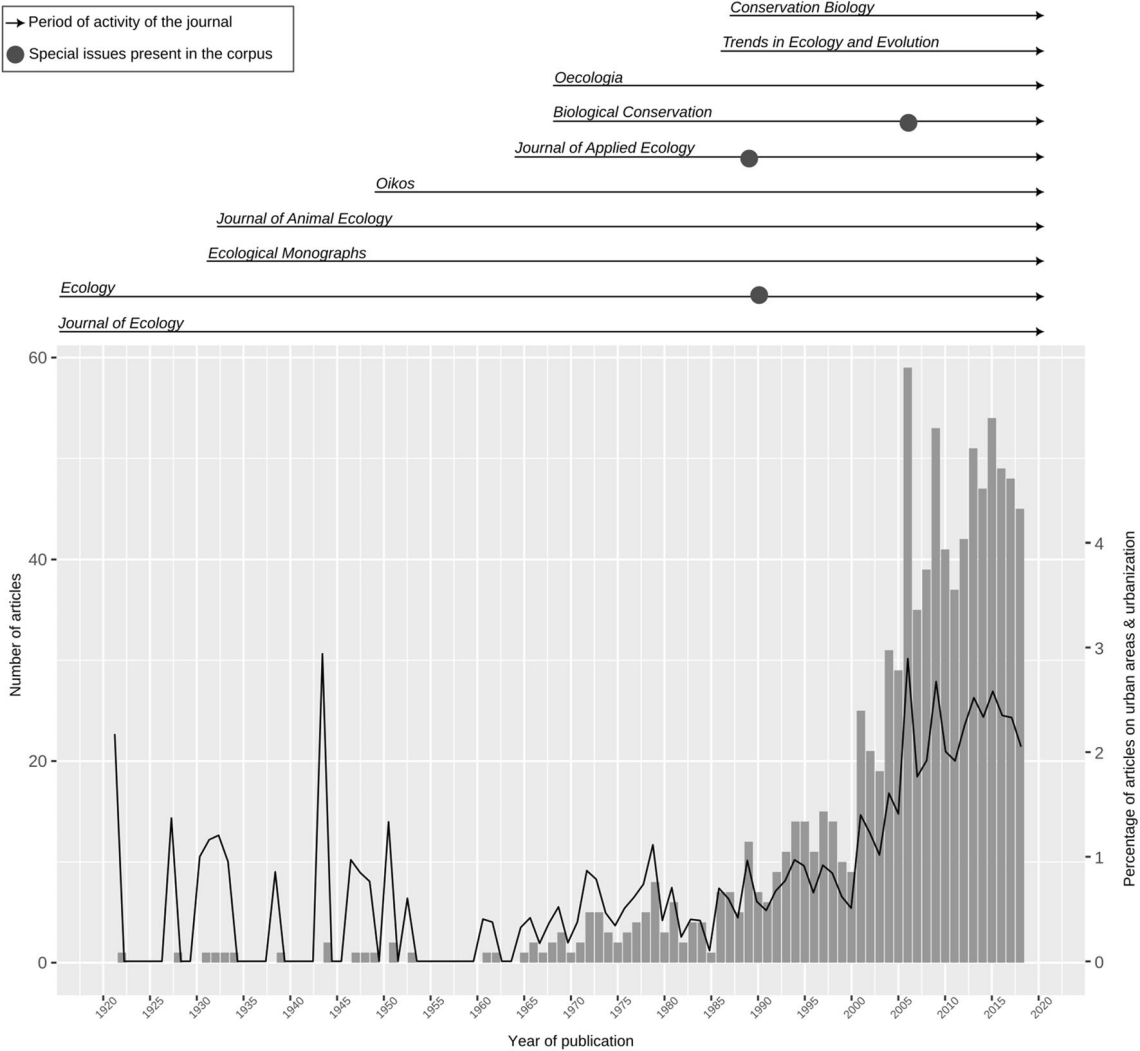
Period of activity of the journals, special issues on urban-related topics present in the corpus, number of papers per year based on the corpus (grey bars), and percentage of papers on urban areas and urbanization (black line), according to the query and selection process explained in the Methods section. The percentage corresponds to the yearly ratio between the number of articles in the corpus and the total number of articles published in the 10 selected journals

According to our corpus, very few papers were published on the urban in ecology before the 1960s (Fig. 1). However, during this period journals generally published only a few issues, and thus only a small number of papers, per year; the percentage of papers included in the corpus was therefore often over 1% for this period. The number of papers on the urban in ecology then increased, but the corpus is characterized by small peaks and troughs, which are essentially coincidental, until the 1990s. However, the 1989 peak corresponds to a special issue on “environmental problems of industrialized countries” in *Journal of Applied Ecology*. From 1990 to 2018, the number of publications per year increased substantially. Some peaks in the corpus are directly related to special issues on cities or urbanization, such as the 1990 special feature on “the use of urban gradients in ecological studies” in *Ecology* (volume 71, issue 4) and the 2006 special issue on urbanization in *Biological Conservation* (volume 127, issue 3). Other peaks cannot be easily explained based on information from the articles themselves.

### Semantic fields of scientific publications on urban areas and urbanization

**Fig. 2:**
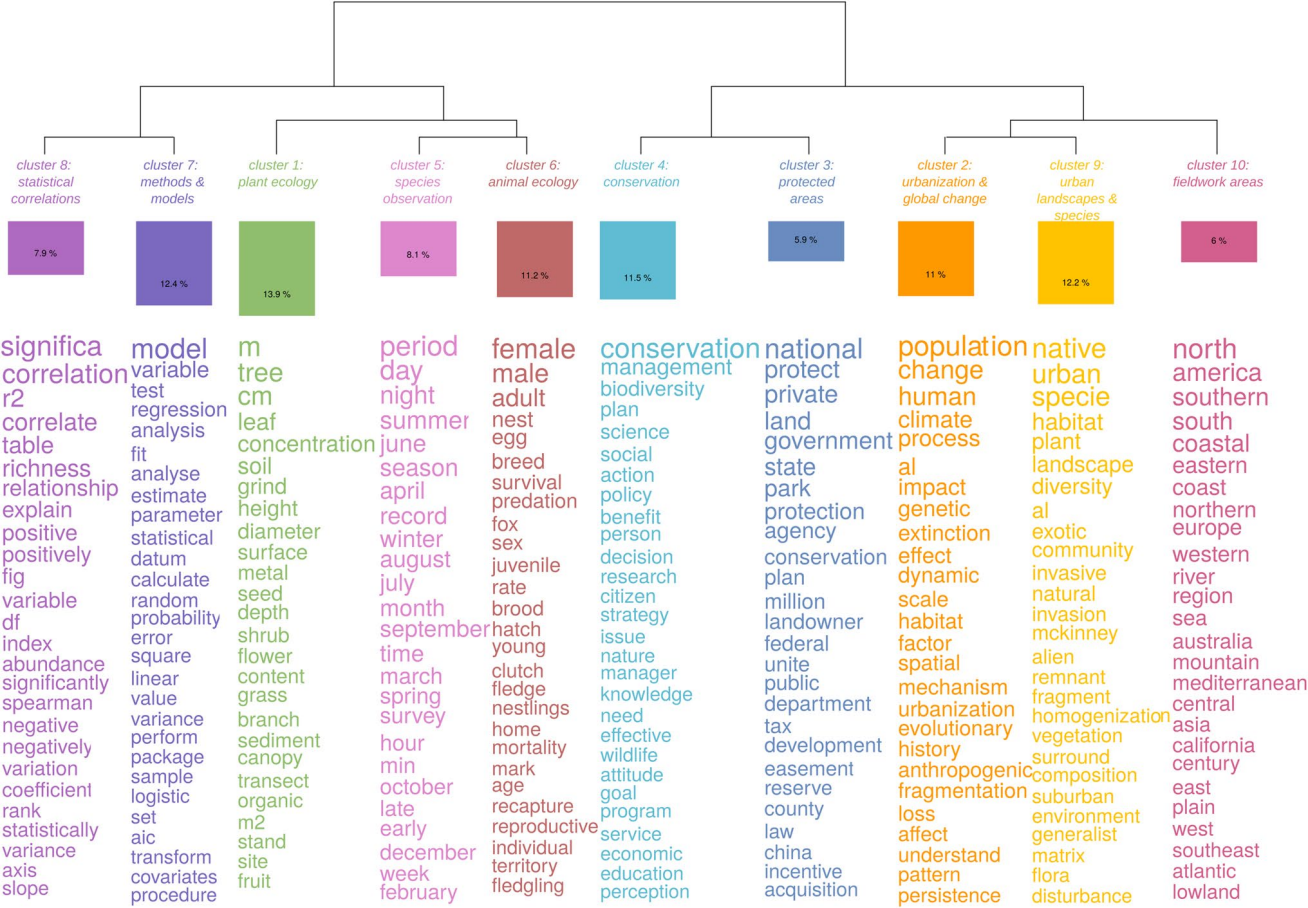
Dendrogram representing the 10 semantic fields of the corpus of scientific publications in ecology (1922–2018). The figure is based on the hierarchical spectral clustering analysis of the textual segments, performed with Iramuteq. For each semantic field and in each cluster, the lemmas are ranked in descending order of degree of association with the clusters (using the signed χ^2^ association metric), also expressed by the size of the text. Keywords, established through a qualitative analysis, are presented under the class numbers to facilitate interpretation

The hierarchical spectral clustering analysis produced 10 clusters corresponding to the major semantic fields of the corpus of scientific articles. These clusters and semantic fields are shown in a dendrogram (Fig. 2) and are characterized by quotations and examples of publications (Supplementary information 1).

- ***Cluster 1*** (13.9% of the text segments) groups text segments and texts relating mostly to plant ecology. Many of the articles representative of this cluster were published before 2000.
- ***Cluster 2*** (11% of the text segments) relates to urbanization and global change, and more generally to major social and environmental changes, such as climate change, species extinction, and habitat fragmentation. This cluster is mostly composed of more recent texts.
- ***Cluster 3*** (5.95% of the text segments) concerns parks and protected areas, a theme strongly present in conservation journals.
- ***Cluster 4*** (11.5% of the text segments) focuses on biodiversity conservation and connections between science and management; as with cluster 3, this theme is very present in articles published in conservation journals.
- ***Cluster 5*** (8.1% of the text segments) comprises text segments containing information on the data collection process and on the observation of different species.
- ***Cluster 6*** (11.2% of the text segments) focuses more generally on animal ecology.
- ***Clusters 7*** and ***8*** (over 20% of the text segments) relate to the description of methods and results in connection with statistical analyses.
- ***Cluster 9*** (12.2% of the text segments) is related to urban habitats and species.
- ***Cluster 10*** (10% of the text segments) groups words and text segments which describe the areas where fieldwork was conducted.

### Changes to the 10 semantic fields over time (1922–2018)

**Fig. 3:**
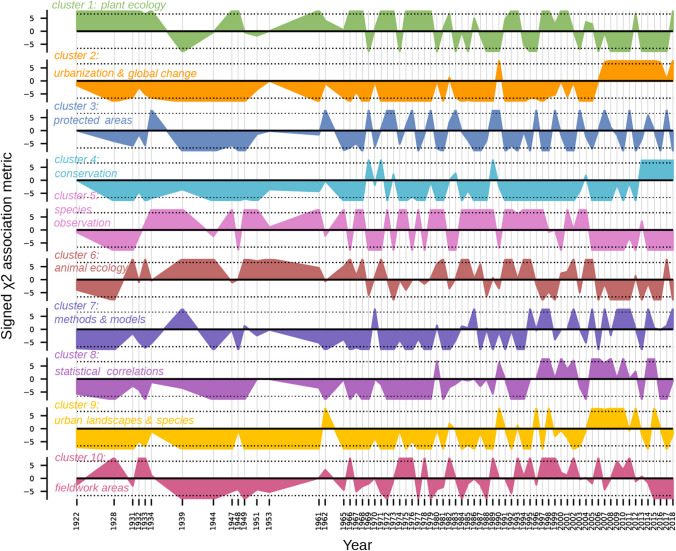
Over- and underrepresentation (distribution above and below the zero line, respectively) of the 10 semantic fields (1922–2018). The scale on the left represents the signed χ^2^ association metric values, and the dotted lines represent the 0.01 significance level for a χ^2^ distribution with 1 degree of freedom (= 6.63). The colours used in this figure are the same as in Fig. 2

Figure 3 shows the distribution of the 10 semantic fields within the corpus over time. It is clear that the semantic fields are not evenly distributed over time. For example, plant ecology (cluster 1) was overrepresented in the corpus from the beginning of the study period to the mid-1930s (signed χ^2^ association metric > 6.63). This semantic field was slightly underrepresented in 1939 (signed χ^2^ association metric < -6.63), before being once again overrepresented in 1947 and 1949 and at the beginning of the 1960s, and then mostly overrepresented from the mid-1960s to the beginning of the 1980s. It was then alternately over- and underrepresented until the mid-2000s, after which point it was essentially underrepresented.

Also, text segments on methods, statistics and modelling (clusters 7 and 8) have become increasingly overrepresented since the mid-1990s, whereas observations and inventories (clusters 5 and 6) have become less common since the mid-2000s. While the semantic field related to protected areas (cluster 3) has regularly been overrepresented throughout the study period (in one year during the 1930s, at the beginning of the 1960s, and more often in recent years), biodiversity conservation (cluster 4) was briefly overrepresented at the end of the 1960s and at the beginning of the 1970s but has mostly gained momentum only since 2012. Urbanization and global change (cluster 2) have also only become overrepresented in the corpus since the mid-2000s, along with urban landscapes and species (cluster 9), despite this semantic field’s underrepresentation in 2014 and 2017.

**Fig. 4:**
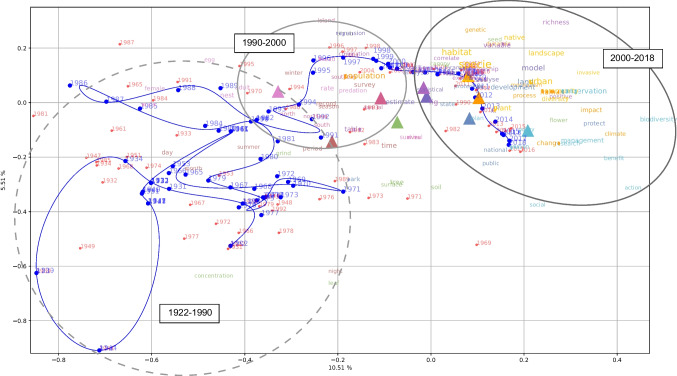
Factorial map representing the correspondence analysis based on the contingency table between lemmas appearing at least 1,000 times in the corpus and publication years (colours in Fig. 4 are the same as in Fig. 2 and 3). The top 10 lemmas for each cluster (based on their χ^2^ associations; Fig. 2) are displayed, with the size of the text corresponding to the frequency in the corpus. The barycentres of the semantic fields (triangles), the publication years (red dots), and the moving average (blue line) are also shown. The horizontal dimension explains 10.51% of the variance, whereas the vertical dimension explains 5.51%

The factorial map (Fig. 4) helps visualize the main lexical contrasts and proximities within the corpus according to the year of publication of the papers. The map illustrates the lexical differences between the pre-2000 publications (on the left side of Fig. 4) and the post-2000s publications (on the right side of Fig. 4). Moreover, the trajectory of the moving average curve before the 1990s (blue line on the left side of Fig. 4) is rather erratic and convoluted. This is in contrast to the curve’s trajectory between the 1990s and 2018, which shows a clear progression (on the far right of Fig. 4). The small number of keywords situated in the pre-1990 ellipsis reveals the extent to which the corpus is unbalanced in the number of papers published before and after 1990 and it highlights the difficulty interpreting pre-1990 changes in vocabulary. Post-1990, many more keywords appear, and the progression towards issues such as biodiversity conservation and climate change can be seen.

## Discussion and conclusion

In this paper, we have investigated the changing importance of the urban in the ecological literature over the past century using bibliometrics and textual data analysis. We aimed to provide a novel contribution to the understanding of the rise of the urban in ecology, and more generally of the relationship between ecology and the urban.

We have opted for journals among the oldest in ecology with broad scopes and with a regular rate of publication. This approach ensured the consistency and homogeneity of the corpus, two conditions which are essential when producing and analyzing text corpora (Pincemin 1999). We have selected eight journals with a broad perspective in ecology and two focused on conservation biology. Our in-depth text data analysis contrasts with previous works (Barot et al. 2019; Young and Wolf 2006) focused on short or medium time spans. Besides, our selection of specific journals at the outset, with respect to a random selection of papers based on keywords (e.g., Barot et al. 2019) has allowed us to limit in number the sample of papers in order to be able to carefully read each of them and exclude non-relevant articles.

By focusing on the urban in ecology, and not solely on research that has been labelled as ‘urban ecology’, our study clearly shows that urban environments were never completely absent from international publications in mainstream ecology journals during the past century. Our results (Fig. 1) show that cities and urbanization have been present for a long time in publications in ecology, but at a very low intensity. Thus, considering the small percentage of literature on cities before the 1990s, our study also corroborates the results from a qualitative review on urban ecology, in which the author states that urban ecology was hardly visible in mainstream ecology journals before the late 1980s (Wu 2014).

Our study helps visualize a first turning point in the 1970s, mentioned in some previous qualitative reviews (Breuste et al. 1998; Sukopp 1998; Douglas and Goode 2010; McDonnell 2015). Based on the textual data analysis (and notably the topics and semantic fields), the growing number of publications on urban areas starting in the 1970s seems to be connected with concerns regarding the spatial expansion of urban areas and discussions on the role protected areas can play in the mitigation of the effects of urbanization.

More strikingly, the quantitative textual data analysis confirms a second turning point in the 1990s, noted in previous qualitative reviews (Parris 2004; Johnston and Daniels 2006; McDonnell et al. 2011). Further, it shows the extent to which publications on the urban were scattered and followed no clear temporal structure before 1990, and it indicates that no leading topics clearly emerged in relation to the urban during that period. We tested the clustering algorithm on a subcorpus comprising the texts spanning from 1922 to 1990 and the resulting clusters were impossible to interpret, revealing the strong heterogeneity of the pre-1990 articles. In this respect, the factorial map (Fig. 4) clearly reveals the contrast between the pre-1990 period, during which the discourse appears much less structured (notably considering the moving average) and the post-1990s periods, when the vocabulary is much more identifiable. Future studies seeking to better understand the pioneering research on the urban in ecology would need to use qualitative methods to identify the main topics or ideas present in publications in ecology in relation to cities and urbanization, including in pre-1990 articles. Some attempts at this task have already been made (Flaminio et al. 2022), but it would be particularly relevant to explore corpora based on non-English language publications. Indeed, many pioneering studies on the urban in ecology were published in other languages (e.g., Kühnelt 1955; Sukopp 1973; Duvigneaud 1974; Kunick 1974; Pyšek 1975), and researchers from non-Anglophone countries and universities have played an important role in the development of research on urban environments (Norra and Petney 2016). Such efforts could corroborate and complement our result that publications on the urban were particularly scattered and unstructured before the 1990s.

Our results show a third turning point in the 2000s, in the form of a major change in topics, semantic fields and vocabulary. Specifically, the topics of urbanization and urban landscapes became overrepresented in the corpus during those years (Fig. 3). Such a change indicates that the emergence of a research field, with papers which regularly bring up planning issues, as well as major environmental issues such as climate change. Environmental concerns have already been put forth in previous studies as a factor contributing to the reinforcement of urban ecology (Cressey 2015). Moreover, the recent overrepresentation of the semantic fields of urban landscapes and urbanization suggests that if the urban was indeed present in articles before 2000, it was most likely less discussed and under-theorized. A similar trend, e.g., the lack of theoretical insights in early studies in ecology focusing on urban areas, has been suggested by previous reviews of urban ecology (Young 2009).

Finally, our paper contributes to the literature on discipline and specialty formation through its experimental approach to decrypting the vocabulary used in the main publications affiliated with a discipline. It privileges a quantitative approach where qualitative discourse approaches often dominate (e.g., Frickel 2004)^1^. Notably, the textual data analysis, which complemented the bibliometric analysis, enabled detection of the stabilization and homogenization of the vocabulary, concomitant to the establishment of a specific research subfield.

This main finding could be tested on other disciplinary or more specific corpora in ecology and biology, for instance on taxa specific journals (e.g., *Journal of Avian Biology*) or on journals dedicated to specific biological processes (e.g., *Biological invasions*^2^) Indeed, this way of scrutinizing the emergence of research subfields, such as urban ecology, through changes in vocabulary seems particularly promising and deserves to be explored more widely. We hope that our proposition will encourage the use of bibliometrics and textual data analysis as complementary tools to better understand the rise of new topics and research fields.

## Supplementary Information

Below is the link to the electronic supplementary material.

Supplementary file1 (DOCX 50.7 KB)

## Data Availability

No datasets were generated or analysed during the current study.

## Footnotes

1 The type of quantitative approach we propose here can be usefully combined with a qualitative approach. Such mixed-methods research design therefore led us to identify different urban imaginaries associated with the city in the history of ecology (Flaminio et al. 2022) as well as to define more specifically what inattention to the city in ecology can mean (Chalmandrier et al. 2024).

2 Interestingly, *Biological Invasions* published a special issue dedicated to urban invasions in 2017.
