# Supplementary material for: The urban in ecology: a quantitative textual analysis of the scientific literature over a century
Source: Urban Ecosyst. 2024 Sep 6;27(6):2531–42. doi: 10.1007/s11252-024-01603-4 (PMC11461683; doi:10.1007/s11252-024-01603-4)
Supplement: Supplementary file 1 — Supplementary file1 (DOCX 50.7 KB) [file 11252_2024_1603_MOESM1_ESM.docx]

**Supplementary information 1**. Examples of text segments, papers and journals characterizing the 10 different clusters and semantic fields.

| Cluster # | Name of semantic field | Examples of text segments attributed to one of the 10 clusters and semantic fields | Examples of papers with over 60% of text segments attributed to the cluster | Journals with over 10% of text segments attributed to the cluster |
| --- | --- | --- | --- | --- |
| 1 | Plant ecology | *“Most of the vegetation on these light soils was probably Quercetum sessiliflorae, but there are also traces of a more heathy type of vegetation. The chalk, which forms the soil of so much of the neighbouring counties, […]”* (Richards, 1928)  *“Grass samples were typically cut from as near the stream as possible, leaves were collected from debris dams within the stream channel, and periphyton samples were collected from within the stream.”* (Newcomer et al., 2012) | (Groet, 1976; Lotschert & Kohm, 1977; Weathers et al., 2001) | *Journal of Ecology; Oecologia; Ecological Monographs; Oikos;*  *Journal of Applied Ecology; Ecology* |
| 2 | Urbanization and global change | *“We found that urbanization induced community homogenization and that populations of specialist species became increasingly unstable with increasing urbanization of the landscape. Our results emphasize that urbanization has a substantial impact on the spatial component of communities and highlight the destabilizing effect of urbanization on communities over time.”* (Devictor et al., 2007)  *“Cities are not simply altering biodiversity by reducing the number and variety of native species. Humans are selective agents determining which species can live in cities and causing organisms to undergo rapid evolutionary change.”* (Alberti, 2015) | (Alberti, 2015; Thompson et al., 2018) | *Trends in Ecology and Evolution; Oikos; Ecology* |
| 3 | Protected areas | *“The government has also called for enhancing conservation in the framework of national policies […], and has invested heavily in improving and expanding protected areas”* (Miller-Rushing et al., 2017)  *“In 1943, the London natural history society set up a committee to prepare a list of proposed nature reserves for the London area. Castell (1947) listed the eight existing reserves, royal parks and metropolitan water board reservoirs and then gave brief descriptions of 93 other sites selected on biological grounds.”* (Davis, 1976) | (Milder & Clark, 2011; Miller-Rushing et al., 2017; Reed et al., 2014; Whitelaw & Eagles, 2007) | *Conservation Biology* |
| 4 | Conservation | *“Within the past few years, public opinion has been focused on the significance of our physical surroundings in the urban as well as in the rural spheres. The ‘man in the street’ is alert and no longer accepts a careless intervention in the countryside.”* (Benthem, 1973)  *“Urbanization creates new challenges for biodiversity conservation. As a large part of the world’s population moves from rural to urban areas, there are changes in the link between human activities and biodiversity, and consequently to the way we should think biodiversity conservation policies.”* (de Oliveira et al., 2011) | (Asah & Blahna, 2013; Ballard et al., 2017; Ferketic et al., 2010; Karimi et al., 2017; Morrison, 2015) | *Conservation Biology*; *Trends in Ecology and Evolution*; *Biological Conservation*; *Journal of Applied Ecology* |
| 5 | Species observation | *“In late September and October some of the mature slugs have exhibited a quite definite old ‘moth-eaten’ appearance which has been taken to foretell their demise in the near future.”* (Barnes & Weil, 1944) | (Da Silva & Kempenaers, 2017; Roth & Lima, 2007) | *Journal of Animal Ecology; Ecology* |
| 6 | Animal ecology | *“The largest clutches (mean 5.3 eggs) were laid […] which in turn laid a higher percentage of 6 and 7 egg clutches than did presumed 1^st^-year birds.”* (Middleton, 1979)  *“We also assessed urban mortality using the data from the rescue campaigns annually conducted in the archipelago. Urban mortality concerned about 6% of fledglings, but its importance greatly varied among islands. When rescue campaigns occur, the rate might drop below 0.5%.”* (Fontaine et al., 2011) | (Aldredge et al., 2014; Husby, 1986; Richner, 1992; Sasvari & Hegyi, 1994) | *Journal of Animal Ecology; Oikos; Oecologia; Ecology; Journal of Applied Ecology* |
| 7 | Methods and models | *“Generalized linear models using a Gaussian distribution were used to investigate the relationship between the total richness of species, matrix-tolerant species remnant-reliant species, ground foraging species, shrub foraging species, canopy foraging species, migrants […]”* (Palmer et al., 2008) | *No paper had over 60% of its text segments attributed to the cluster* | *Ecological Monographs; Journal of Animal Ecology; Oikos; Journal of Applied Ecology* |
| 8 | Statistical correlation | *“Possibility of differences between 13- and 8-year-old populations was tested with analysis of variance for species number, numbers of individuals and community diversity. In each case, significant variation was between collections from stations of equal age, not between stations of different age.”* (Lussenhop, 1973) | *No paper had over 60% of its text segments attributed to the cluster* | *Oikos* |
| 9 | Urban species | *“The presence of umbrella species is often indicative of high taxonomic diversity; however, functional diversity is now recognized as an important metric for biodiversity and thus should be considered when choosing umbrella species.”* (Sattler et al., 2014) | (Kowarik & von der Lippe, 2018; McKinney, 2006; Williams et al., 2009) | *Trends in Ecology and Evolution; Journal of Ecology; Biological Conservation; Journal of Applied Ecology* |
| 10 | Fieldwork areas | *“The Swiss plateau is about 30% of the surface of Switzerland (approximately 12 500 km^2^) and is characterized by intensive agriculture, managed forests, and densely populated urban areas (380 people/km^2^).”* (Sattler et al., 2014) | *No paper had over 60% of its text segments attributed to the cluster* | *No journal had over 10% of its text segments attributed to the cluster* |

**References**

Alberti M (2015) Eco-evolutionary dynamics in an urbanizing planet. Trends in Ecology & Evolution 30:114–126. https://doi.org/10.1016/j.tree.2014.11.007

Aldredge RA, Boughton RK, Rensel MA, et al (2014) Hatching asynchrony that maintains egg viability also reduces brood reduction in a subtropical bird. Oecologia 174:77–85. https://doi.org/10.1007/s00442-013-2749-x

Asah ST, Blahna DJ (2013) Practical Implications of Understanding the Influence of Motivations on Commitment to Voluntary Urban Conservation Stewardship. Conservation Biology 27:866–875. https://doi.org/10.1111/cobi.12058

Ballard HL, Robinson LD, Young AN, et al (2017) Contributions to conservation outcomes by natural history museum-led citizen science: Examining evidence and next steps. Biological Conservation 208:87–97. https://doi.org/10.1016/j.biocon.2016.08.040

Barnes HF, Weil JW (1944) Slugs in Gardens: Their Numbers, Activities and Distribution. Part I. Journal of Animal Ecology 13:140–175. https://doi.org/10.2307/1449

Benthem RJ (1973) Recreational and environmental planning. Biological Conservation 5:1–5. https://doi.org/10.1016/0006-3207(73)90045-1

Da Silva A, Kempenaers B (2017) Singing from North to South: Latitudinal variation in timing of dawn singing under natural and artificial light conditions. Journal of Animal Ecology 86:1286–1297. https://doi.org/10.1111/1365-2656.12739

Davis BNK (1976) Wildlife, urbanisation and industry. Biological Conservation 10:249–291. https://doi.org/10.1016/0006-3207(76)90002-1

de Oliveira JAP, Balaban O, Doll CNH, et al (2011) Cities and biodiversity: Perspectives and governance challenges for implementing the convention on biological diversity (CBD) at the city level. Biological Conservation 144:1302–1313. https://doi.org/10.1016/j.biocon.2010.12.007

Devictor V, Julliard R, Couvet D, et al (2007b) Functional homogenization effect of urbanization on bird communities. Conservation Biology 21:741–751. https://doi.org/10.1111/j.1523-1739.2007.00671.x

Ferketic JS, Latimer AM, Silander JA (2010) Conservation justice in metropolitan Cape Town: A study at the Macassar Dunes Conservation Area. Biological Conservation 143:1168–1174. https://doi.org/10.1016/j.biocon.2010.02.024

Fontaine R, Gimenez O, Bried J (2011) The impact of introduced predators, light-induced mortality of fledglings and poaching on the dynamics of the Cory’s shearwater (Calonectris diomedea) population from the Azores, northeastern subtropical Atlantic. Biological Conservation 144:1998–2011. https://doi.org/10.1016/j.biocon.2011.04.022

Groet SS (1976) Regional and Local Variations in Heavy Metal Concentrations of Bryophytes in the Northeastern United States. Oikos 27:445–456. https://doi.org/10.2307/3543463

Husby M (1986) On the Adaptive Value of Brood Reduction in Birds: Experiments with the Magpie Pica pica. Journal of Animal Ecology 55:75–83. https://doi.org/10.2307/4693

Karimi A, Tulloch AIT, Brown G, Hockings M (2017) Understanding the effects of different social data on selecting priority conservation areas. Conservation Biology 31:1439–1449. https://doi.org/10.1111/cobi.12947

Kowarik I, von der Lippe M (2018) Plant population success across urban ecosystems: A framework to inform biodiversity conservation in cities. Journal of Applied Ecology 55:2354–2361. https://doi.org/10.1111/1365-2664.13144

Lotschert W, Kohm H (1977) Characteristics of Tree Bark as an Indicator in High-Immission Areas. Oecologia 27:47–64. https://doi.org/10.1007/BF00345684

Lussenhop J (1973) The Soil Arthropod Community of a Chicago Expressway Margin. Ecology 54:1124–1137. https://doi.org/10.2307/1935579

McKinney ML (2006) Urbanization as a major cause of biotic homogenization. Biological Conservation 127:247–260. https://doi.org/10.1016/j.biocon.2005.09.005

Middleton ALA (1979) Influence of Age and Habitat on Reproduction by the American Goldfinch. Ecology 60:418–432. https://doi.org/10.2307/1937669

Milder JC, Clark S (2011) Conservation Development Practices, Extent, and Land-Use Effects in the United States. Conservation Biology 25:697–707. https://doi.org/10.1111/j.1523-1739.2011.01688.x

Miller-Rushing AJ, Primack RB, Ma K, Zhou Z-Q (2017) A Chinese approach to protected areas: A case study comparison with the United States. Biological Conservation 210:101–112. https://doi.org/10.1016/j.biocon.2016.05.022

Morrison SA (2015) A framework for conservation in a human-dominated world. Conservation Biology 29:960–964. https://doi.org/10.1111/cobi.12432

Newcomer TA, Kaushal SS, Mayer PM, et al (2012) Influence of natural and novel organic carbon sources on denitrification in forest, degraded urban, and restored streams. Ecological Monographs 82:449–466. https://doi.org/10.1890/12-0458.1

Palmer GC, Fitzsimons JA, Antos MJ, White JG (2008) Determinants of native avian richness in suburban remnant vegetation: Implications for conservation planning. Biological Conservation 141:2329–2341. https://doi.org/10.1016/j.biocon.2008.06.025

Reed SE, Hilty JA, Theobald DM (2014) Guidelines and Incentives for Conservation Development in Local Land-Use Regulations. Conservation Biology 28:258–268. https://doi.org/10.1111/cobi.12136

Richards PWM (1928) Ecological Notes on the Bryophytes of Middlesex. Journal of Ecology 16:269–300. https://doi.org/10.2307/2255804

Richner H (1992) The Effect of Extra Food on Fitness in Breeding Carrion Crows. Ecology 73:330–335. https://doi.org/10.2307/1938744

Roth TC, Lima SL (2007) The predatory behavior of wintering Accipiter hawks: temporal patterns in activity of predators and prey. Oecologia 152:169–178. https://doi.org/10.1007/s00442-006-0638-2

Sasvari L, Hegyi Z (1994) Colonial and Solitary Nesting Choice as Alternative Breeding Tactics in Tree Sparrow Passer-Montanus. Journal of Animal Ecology 63:265–274. https://doi.org/10.2307/5545

Sattler T, Pezzatti GB, Nobis MP, et al (2014) Selection of Multiple Umbrella Species for Functional and Taxonomic Diversity to Represent Urban Biodiversity. Conservation Biology 28:414–426. https://doi.org/10.1111/cobi.12213

Thompson KA, Rieseberg LH, Schluter D (2018) Speciation and the City. Trends in Ecology & Evolution 33:815–826. https://doi.org/10.1016/j.tree.2018.08.007

Weathers KC, Cadenasso ML, Pickett STA (2001) Forest edges as nutrient and pollutant concentrators: Potential synergisms between fragmentation, forest canopies, and the atmosphere. Conservation Biology 15:1506–1514. https://doi.org/10.1046/j.1523-1739.2001.01090.x

Whitelaw GS, Eagles PFJ (2007) Planning for long, wide conservation corridors on private lands in the Oak Ridges Moraine, Ontario, Canada. Conservation Biology 21:675–683. https://doi.org/10.1111/j.1523-1739.2007.00708.x

Williams NSG, Schwartz MW, Vesk PA, et al (2009) A conceptual framework for predicting the effects of urban environments on floras. Journal of Ecology 97:4–9. https://doi.org/10.1111/j.1365-2745.2008.01460.x
